# Structural Insight into KsBcl-2 Mediated Apoptosis Inhibition by Kaposi Sarcoma Associated Herpes Virus

**DOI:** 10.3390/v14040738

**Published:** 2022-03-31

**Authors:** Chathura D. Suraweera, Mark G. Hinds, Marc Kvansakul

**Affiliations:** 1 Department of Biochemistry and Chemistry, La Trobe Institute for Molecular Science, La Trobe University, Bundoora, VIC 3086, Australia; chathura.suraweera@monash.edu; 2 Bio21 Molecular Science and Biotechnology Institute, The University of Melbourne, Parkville, VIC 3052, Australia

**Keywords:** Bcl-2, apoptosis, Bid, Kaposi Sarcoma Herpesvirus, herpesviruses, X-ray crystallography

## Abstract

Numerous large DNA viruses have evolved sophisticated countermeasures to hijack the premature programmed cell death of host cells post-infection, including the expression of proteins homologous in sequence, structure, or function to cellular Bcl-2 proteins. Kaposi sarcoma herpes virus (KSHV), a member of the *gammaherpesvirinae*, has been shown to encode for KsBcl-2, a potent inhibitor of Bcl-2 mediated apoptosis. KsBcl-2 acts by directly engaging host pro-apoptotic Bcl-2 proteins including Bak, Bax and Bok, the BH3-only proteins; Bim, Bid, Bik, Hrk, Noxa and Puma. Here we determined the crystal structures of KsBcl-2 bound to the BH3 motif of pro-apoptotic proteins Bid and Puma. The structures reveal that KsBcl-2 engages pro-apoptotic BH3 motif peptides using the canonical ligand binding groove. Thus, the presence of the readily identifiable conserved BH1 motif sequence “NWGR” of KsBcl-2, as well as highly conserved Arg residue (R86) forms an ionic interaction with the conserved Asp in the BH3 motif in a manner that mimics the canonical ionic interaction seen in host Bcl-2:BH3 motif complexes. These findings provide a structural basis for KSHV mediated inhibition of host cell apoptosis and reveal the flexibility of virus encoded Bcl-2 proteins to mimic key interactions from endogenous host signalling pathways.

## 1. Introduction

Programmed cell death forms part of a suite of immune response strategies utilized by multicellular organisms to eliminate invading pathogens [1]. In order to counter host cell death-based defence mechanisms viruses have co-evolved genes to either counter or enhance host programmed cell death to ensure their own proliferation and survival [2]. One such strategy for viral manipulation of the host-cell programmed cell death response has been the acquisition of pro-survival homologs of the B-cell lymphoma 2 (Bcl-2) family that mediate intrinsic or mitochondrial initiated apoptosis [3]. Bcl-2 proteins are critical arbiters of intrinsic apoptosis and are characterized by the presence of one or more conserved Bcl-2 homology (BH) sequence motifs and a unique Bcl-2 fold, though the viral Bcl-2 homologs frequently lack any sequence conservation with their mammalian counterparts, the protein fold is maintained [4,5].

The mammalian Bcl-2 family can be divided into pro-survival or pro-apoptotic members. Mammalian pro-survival members comprise Bcl-2, Bcl-x_L_, Mcl-1, Bcl-w, A1 and Bcl-B all of which harbor multiple BH motifs in addition to a C-terminal transmembrane region that targets them to the outer mitochondrial membrane [4,6]. The other members of the family are pro-apoptotic Bcl-2 proteins, and these are subdivided into two groups: those that feature multiple BH motifs such as Bak, Bax and Bok; and others that contain only the BH3 motif and are denoted as BH3-only proteins [7]. The mammalian BH3-only proteins include Bad, Bid, Bik, Bim, Bmf, Hrk, Noxa and Puma, and act either by directly activating Bak and Bax, or they counter the ability of pro-survival Bcl-2 proteins to hold Bak and Bax in check [8]. A number of herpes viruses have acquired pro-survival Bcl-2 homologs among their survival genes [9], such as Epstein–Barr virus (EBV) encoded BHRF-1 [10,11] and BALF-1 [12], Kaposi-sarcoma associated herpesvirus (KSHV) apoptosis regulator KsBcl-2 [13], Herpesvirus saimiri Bcl-2 homolog HVS-1 ORF16 [14], Turkey herpesvirus encoded HVT079 [15,16] and murine gammaherpesvirus 68 encoded M11 [17].

Interactions between pro-survival Bcl-2 proteins and the BH3 motif of pro-apoptotic proteins occur via a conserved hydrophobic ligand binding groove on the pro-survival Bcl-2 protein neutralising their pro-survival activity allowing pro-apoptotic Bcl-2 proteins to initiate apoptosis. Key contributors to the cell death are the pro-apoptotic Bax and Bak proteins. After activation, Bax and Bak multimerize on the mitochondrial outer membrane to form large pores [18] that results in the release of pro-apoptotic factors including cytochrome *c*. Cytochrome *c* together with the adapter protein APAF-1 forms the apoptosome [19], which provides a platform for initiator caspases and enables activation of downstream executioner caspases and subsequent dismantling of the cell. The presence of pro-survival viral Bcl-2 homologs inhibits the action of Bax and Bak to preserve the cell for viral replication [20].

KSHV or human herpesvirus-8 (HHV8) is an oncogenic virus of the *herpesviridae* family belonging to the subfamily *gammaherpesvirinae* and genus rhadinovirus. Other members of rhadinovirus include the closely related herpesvirus saimiri as well as murine gammaherpesvirus 68 and Macaca mulatta rhadinovirus. While oncogenic EBV belongs to the same subfamily of *gammaherpesvirinae*, it is not a member of the genus rhadinovirus. KSHV primarily infects host B-lymphocytes and endothelial cells causing a lifelong latent infection in humans. KSHV is a large linear double stranded DNA virus whose genome size is about ~165 kbp [21,22] that encodes a number of immunomodulatory genes [22,23] including a homolog of the cellular Bcl-2 protein, KsBcl-2 [13,24]. KsBcl-2 lacks any significant shared sequence identity with mammalian Bcl-2 proteins [13] though it does contain a recognizable “NWGR” sequence motif in the BH1 region, which is crucial for pro-survival function of majority of Bcl-2 family proteins. The highly conserved arginine of the “NWGR” sequence makes contact with the BH3 motif of pro-apoptotic Bcl-2 proteins. However, this interaction does not lead to heterodimerization with pro-apoptotic Bcl-2 proteins Bak and Bax at the mitochondrial outer membrane [13]. Furthermore, the *ksbcl-2 [orf16]* gene is expressed in the viral lytic replication cycle [13] and inhibits both cellular apoptosis and autophagy when transiently expressed in cells as well as in in-vitro studies [25,26], similar to that previously observed for murine gammaherpesvirus 68 protein M11 [17,25,26]. In addition, KsBcl-2 is an essential element in KSHV replication, and the virus cannot complete its lytic replication cycle, proliferate and reactivate when mutated [27,28,29]. Moreover, KsBcl-2 is important for virion assembly via tegument protein (ORF55) through its N-terminal amino acid residues 11–20 [30]. Here, we report the structural and biochemical characterization of KSHV encoded KsBcl-2, and its potential role in modulating apoptosis in humans. KsBcl-2 shares 17% sequence identity with its EBV homolog BHRF-1. KsBcl-2 is able to bind to peptides spanning the BH3 motif of human pro-apoptotic Bcl-2 proteins including those of Bax, Bak, Bok, Bim, Bid, Bik, Hrk, Noxa and Puma with a wide range of affinities from nanomolar to sub-micromolar range. We also show KsBcl-2 adopts a globular conserved monomeric Bcl-2-fold structure similar to other characterized herpes virus encoded Bcl-2 homologs and binds BH3 motif peptides using the canonical Bcl-2 ligand binding groove. Combined, these findings establish KsBcl-2 as a potent inhibitor of host pro-apoptotic proteins.

## 2. Materials and Methods

Synthetic cDNA codon optimized for *Escherichia coli* encoding for KsBcl-2 mutant V117A from the Kaposi’s sarcoma-associated herpesvirus lacking the C-terminal 29 residues (spanning residues 1–146) was cloned into the bacterial expression vector pGEX-6P3 (Genscript) and transformed into *E. coli* Codon plus (RIL). Cells were grown in 2YT medium containing 1 μg mL^−1^ ampicillin at 37 °C in a shaker incubator until an OD_600_ of 0.6 was reached, and KsBcl-2 expression was induced by adding isopropyl β-D-1-thiogalactopyranoside (IPTG) to a final concentration of 0.5 mM for 20 h at 18 °C. Cells were harvested by ultracentrifugation at 5000 rpm (JLA 9.1000 rotor, Beckman Coulter Avanti J-E) for 20 min prior to resuspension in 100 mL lysis buffer A (50 mM Tris pH 8.0, 300 mM NaCl and 5 mM DTT (dithiothreitol)). Harvested cells were lysed via sonication (Model 705 Sonic Dismembrator, Fisher Scientific, Hampton, NH, USA) with 6 s pulses up to 1.5 min with 1 min pulse off time between each pulse at an amplitude of 60. The resultant lysate was transferred to SS34 tubes for centrifugation at 18,000 rpm (JA-25.50 rotor, Beckman Coulter Avanti J-E) for 30 min. The supernatant was decanted and loaded onto 2 mL of Glutathione Sepharose 4B resin in a gravity flow column (GE Healthcare) equilibrated with buffer A. Following sample application, the column was washed with 100 mL of buffer A followed by HRV 3C protease cleavage overnight at 4 °C in the presence of 25 mL of buffer A (Addition of excess buffer is essential to stop the after-cleavage aggregation of protein). The liberated target protein was eluted using 30 mL of buffer A and concentrated to a 1.0 mg mL^−1^ using a centrifugal concentrator with a 3 kDa molecular weight cut-off (Amicon^®^ Ultra 15). Concentrated KsBcl-2 protein was subjected to multiple size-exclusion chromatography runs using a Superdex S75 16/60 column mounted on an ÄKTAExpress system (GE Healthcare) equilibrated in 25 mM Tris-HCl pH 8.5, 200 mM NaCl, 5 mM TCEP (Tris(2-carboxyethyl)phosphine hydrochloride), where it eluted as a single peak in the volume corresponding to a monomeric species. The final sample purity was estimated to be higher than 95% based on SDS–PAGE analysis.

### 2.1. Measurement of Dissociation Constants

All affinity measurements were performed in triplicate. Protein concentrations were measured using UV spectrophotometer (Nanodrop, Thermo Scientific) at a wavelength of 280 nm. Peptide concentrations were calculated based on dry peptide weight after synthesis. BH3 domain peptides used were commercially synthesized using liquid phase peptide synthesis (Genscript) and purified to a final purity of 95%, and comprised the following sequences [31]: hsBim (UniProtID: O43521-3 51-DMRPEIWIAQELRRIGDEFNAYYARR-76), hsBak (UniProtID: Q16611-1 67-PSSTMGQVGRQLAIIGDDINRRYDSE-92), hsBax (UniProtID: Q07812-1 50-VPQDASTKKLSECLKRIGDELDSNMELQ-77), hsPuma (UniProtID: Q9BXH1-1 130-EEQWAREIGAQLRRMADDLNAQYERR-155), hsNoxa (UniProtID: Q13794-1 18-PAELEVECATQLRRFGDKLNFRQKLL-43), hsBad (UniProtID: Q92934-1 103-NLWAAQRYGRELRRMSDEFVDSFKKG-128), hsBid (UniProtID: P55957-179-SESQEDIIRNIARHLAQVGDSMDRSIPPGLVNGL-104), hsBik (UniProtID: Q13323-1 51-MEGSDALALRLACIGDEMDVSLRAP-75), hsHrk (UniProtID: O00198-1 26-RSSAAQLTAARLKAIGDELHQRTMWR-51), hsBmf (UniProtID: Q96LC9-1 125-QHQAEVQIARKLQCIADQFHRLHVQQ-151), hsBok: 59-VPGRLAEVCAVLLRLGDELE MIRPSV-84 (UniProtID Q9UMX3). Solution competition assays measuring dissociation constants using a T200 Biacore optical biosensor using BH3 domain peptides and 10 nM KsBcl-2 were performed as previously described [32] and are summarized in Table 1. Assays were performed at room temperature with 0.15 M NaCl, 3 mM EDTA, 0.005% Surfactant P20, 5% (*v*/*v*) DMSO, 0.01 M Hepes pH 7.4 as the running buffer. Bim BH3 peptide was immobilized on CM5 sensorchips using amine-coupling chemistry. Recombinant KsBcl-2 (10 nM) was pre-incubated on ice with varying concentrations of competitor BH3 peptides then injected at the flow rate of 10 uL/min to determine IC_50_ values. All BH3 domain peptides were prepared in running buffer.

### 2.2. Crystallization and Data Collection

A complex of KsBcl-2 with Bid BH3 was reconstituted as previously described [33] by adding Bid BH3-motif peptide at a 1:1.25 molar ratio to KsBcl-2. The reconstituted complex was concentrated to 4.5 mg mL^−1^ using a centrifugal concentrator with a 3 kDa molecular weight cut-off (Amicon^®^ Ultra 0.5). Crystals of KsBcl-2: Bid BH3 were obtained at a protein concentration of 4.5 mg mL^−1^ using the sitting-drop method at 20 °C in 0.1 M Sodium acetate pH 4.6, 8% *w*/*v* PEG 4000. The crystals were KsBcl-2: Bid BH3complex and the hexagonal shaped crystals belonging to space group P6_4_ with a = 89.42 Å, b = 89.42 Å, c = 50.98 Å, α = 90.00 Å, β = 90.00 Å, γ = 120.00 Å in the hexagonal crystal system. Diffraction data were collected on the MX2 beamline at the Australian Synchrotron using an EIGER 16 M detector at a wavelength of 0.9537 Å and an oscillation range of 0.1° per frame [34]. Diffraction data were integrated using DIALS [35] and scaled using AIMLESS [36]. Crystals of KsBcl-2 with Bid BH3 contained one chain of KsBcl-2 and one chain of Bid BH3 in the asymmetric unit, with a calculated solvent content of 46.12%. The structure was phased by molecular replacement using an alphafold [37] generated model for KsBcl-2 as a search model. The final KsBcl-2:Bid BH3 complex was manually built using Coot [38] and refined using PHENIX [39] with final R_work_/R_free_ of 20.1/22.9%, with 98.1% of residues in Ramachandran favoured region of the plot and no outliers.

KsBcl-2: Puma BH3 crystals were grown as described above for the KsBcl-2: Bid BH3 complex. Crystals were obtained in 0.2 M sodium bromide, 0.1 M Bis-tris propane pH 6.5, 20% PEG 3350. The crystals were flash cooled at −173 °C in mother liquor supplemented with 20% ethylene glycol as a cryo-protectant. The KsBcl-2: Puma BH3 complex formed single thin long needle shaped (20 um, 2 um, 1 um) crystals belong to space group C2 with a = 59.22 Å, b = 48.52 Å, c = 57.38 Å, α = 90.00 Å, β = 97.06 Å, γ = 90.00 Å in the monoclinic crystal system. Diffraction data collection, integration and scaling were performed as described above. The molecular replacement was carried out using PHASER with the previously solved structure of KsBcl-2:Bid BH3 as a search model. KsBcl-2: Puma BH3 crystals contain one molecule of KsBcl-2 and one Puma BH3 peptide, with 39.3% solvent content and final TFZ and LLG values of 13.6 and 276 respectively. The final model of KsBcl-2: Puma BH3 was built manually over several cycles using Coot and refined using PHENIX with final R_work_/R_free_ of 23.8/26.6% and 98.3% of residues in Ramachandran favoured region of the plot and no outliers. Details of the data-collection and refinement statistics are summarized in Table 2. All images were generated using PyMOL. All software was accessed via SBGrid [40]. Raw images were deposited with the SBGrid Data Bank [41].

### 2.3. Sequence Search, Alignment, and Interface Analysis

Sequence alignments were performed using MUSCLE [42] with the default settings, and sequence identities were calculated based on the total number of conserved residues in KsBcl-2 against the full sequence. Protein sequences were sourced from Uniprot and have the following accession codes: KSHV KsBcl-2 (UniProt accession number Q76RI8), human Mcl-1 (UniProt Q07820) EBV BHRF1 (UniProt P03182), Zebrafish pro-survival Bcl-2 protein NRZ1 (UniProt Q8UWD5). Protein interfaces were analysed using PISA [43].

### 2.4. Dali 3D Structure Analysis

To identify structural homologs of KsBcl-2, we performed a full search using the Dali webserver (http://ekhidna2.biocenter.helsinki.fi/dali/) [44].

## 3. Results

**Figure 1:**
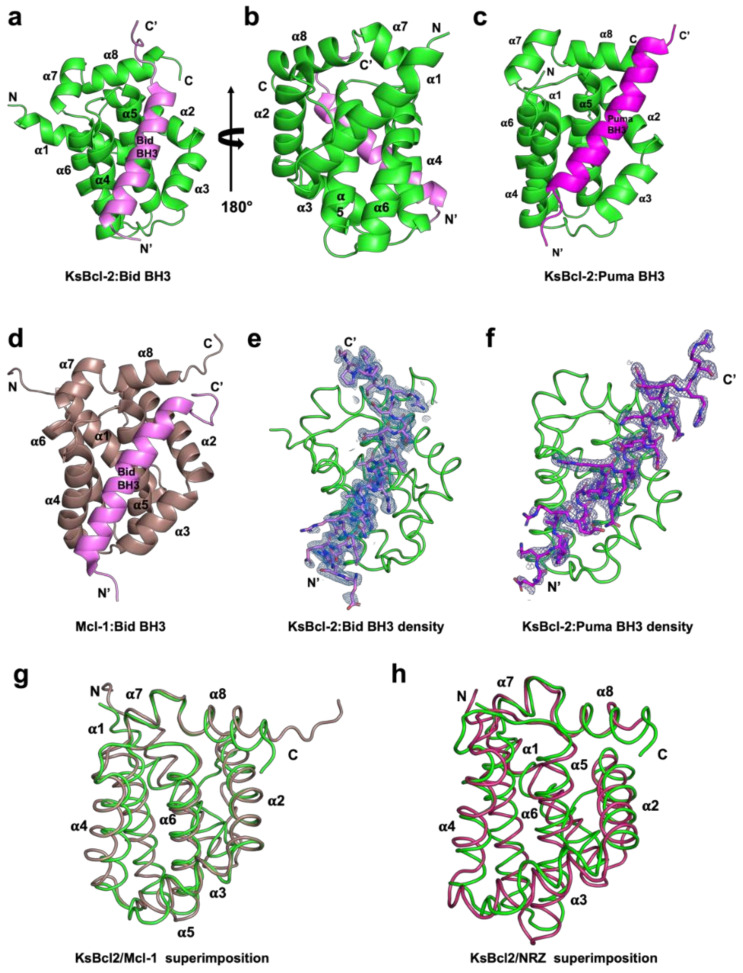
KsBcl-2 binds BH3 motif peptides of pro-apoptotic Bcl-2 proteins using the canonical ligand binding groove. Crystal structures of KsBcl-2 bound to Bid and Puma BH3 motifs. (**a**) KsBcl-2 (green) in complex with the Bid BH3 motif (magenta). KsBcl-2 helices are labelled α1–α8. The view is of the hydrophobic binding groove of one protomer formed by helices α3–α5, and (**b**) is the viewed along the 2-fold symmetry axis (rotated 180 degrees). (**c**) KsBcl-2 (green) in complex with the Puma BH3 domain (magenta) (**d**) human Mcl-1 (raspberry) in complex with the Bid BH3 motif (magenta). (**e**) 2Fo-Fc polder electron density map of KsBcl-2: Bid where electron density for Bid BH3 motif peptide shown in blue interfaces contoured at 1.5·σ (**f**) 2Fo-Fc polder electron density map of KsBcl-2: Puma complex shown as similar to (**d**). (**g**) structural superimposition of backbone of KsBcl-2 (green) onto human Mcl-1 (raspberry). The view is into the canonical hydrophobic binding groove formed by α2–α5. (**h**) structural superimposition of backbone of KsBcl-2 (green) onto zebrafish pro-survival Bcl-2 protein NRZ (warm pink) Images were generated using the PYMOL Molecular Graphics System, Version 1.8 Schrodinger, LLC. 6.

**Figure 2:**
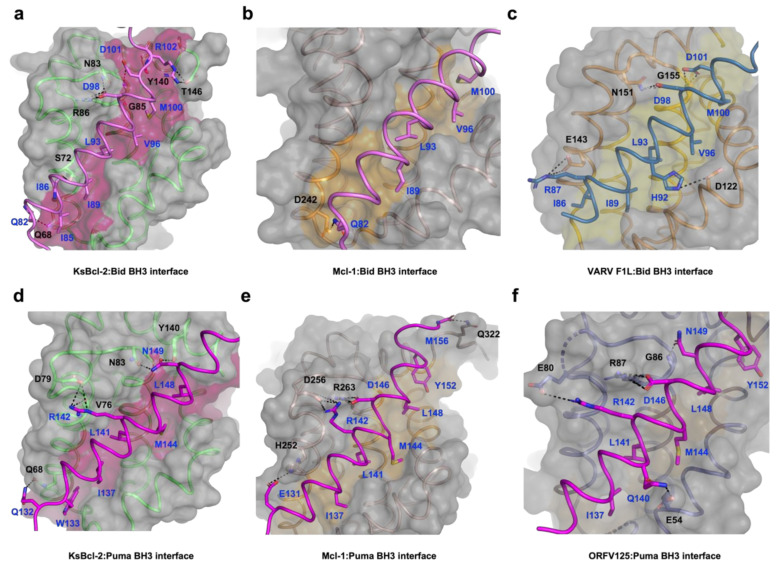
Detailed view of the KsBcl-2:Bid BH3, KsBcl-2:Puma BH3, Mcl1:Bid BH3 and Mcl1:Puma BH3 interfaces. The KsBcl-2 surface, backbone and floor of the binding groove are shown in grey, green and hot pink, respectively. (**a**) KsBcl-2: Bid BH3 interface where Bid BH3 is shown in pink. The five key hydrophobic residues of Bid BH3 (I85, I89, L93, V96 and M100) protruding into the binding groove and the conserved salt-bridge formed by KsBcl-2 R86 and Bid BH3 D98 are labelled, as well as all other residues involved in additional ionic interactions and hydrogen bonds. Interactions are denoted as dashed black lines. (**b**) Molecular surface of human Mcl-1 (brown ribbon): Bid (pink) BH3 is shown as in (**a**). The four key hydrophobic residues of Bid BH3 (I89, L93, V96 and M100) are protruding into the binding groove, and the residues involved in hydrogen bonds are labelled. Interactions are denoted as black dotted lines. (**c**) VARV F1L (orange):Bid BH3 (pink) with the surface of VARV F1L is shown as in (**a**) and the floor of the binding grove is shown in yellow. The five key hydrophobic residues of Bid BH3 (I86, I89, L93, V96 and M100) are protruding into the binding groove and the salt bridge formed by Bid R87 and VARV F1L E143 is labelled, as well as residues involved in hydrogen bonding. (**d**) KsBcl-2: Puma BH3 with the surface of KsBcl-2 is shown as in (**a**), and Puma BH3 is shown in magenta. The five key hydrophobic residues of Puma BH3 (W133, I137, L141, M144 and L148) are protruding into the binding groove and the salt bridge formed by Puma R142 and KsBcl-2 D79 is labelled, as well as residues involved in hydrogen bonds. (**e**) Human Mcl-1 (brown):Puma BH3 is shown as in (**b**). The five key hydrophobic residues of Puma BH3 (I137, L141, M144, L148 and Y152) are protruding into the binding groove, and the conserved salt bridge formed by Puma D146 and Mcl-1 R263 is labelled, as well as residues involved in hydrogen bonds. (**f**) ORFV125 (purple):Puma BH3 is shown as in (**b**). The five key hydrophobic residues of Puma BH3 (I137, L141, M144, L148 and Y152) are protruding into the binding groove, and the conserved salt bridge formed by Puma D146 and ORFV125 R87 is labelled, as well as residues involved in hydrogen bonds. Interactions are denoted as black dotted lines.

To determine whether KSHV KsBcl-2 is able to interfere with Bcl-2 mediated apoptosis by engaging host proapoptotic Bcl-2, we expressed and purified recombinant KsBcl-2 lacking the C-terminal 29 residues comprising the transmembrane motif. We then examined the ability of recombinant KsBcl-2 to bind BH3 motif peptides from all human proapoptotic Bcl-2 proteins using surface plasmon resonance (SPR). SPR revealed a broad interaction spectrum for KsBcl-2 with nanomolar affinities for BH3 motifs from human Bax, Bak, Bok, Bim, Bid, Bik, Noxa, Puma and Hrk with affinities for the BH3-motifs from Bad and Bmf are higher than the 2 mM limit of detection (Table 1). A comparison of the relative affinities of BH3-peptides for KsBcl-2 and the closely related EBV BHRF1 [45], cellular homologue Mcl-1 [46] and fish homologue NRZ [47] is given in Table 3. KsBcl-2 has a much broader spectrum of BH3 binding than EBV BHRF-1 and a similar BH3-binding spectrum as Mcl-1 and NRZ. To establish the structural basis for BH3 ligand engagement by KsBcl-2 we then determined the crystal structure of KsBcl-2 complexes with two of the identified high affinity interactors, Bid and Puma BH3 (Figure 1). The KsBcl-2:Bid complex was refined to 1.41 Å, whereas the KsBcl-2:Puma complex was refined to 2.11 Å. Clear and continuous electron density was observed for KsBcl-2 residues 1–146 and Bid residues 79–104 in the KsBcl-2: Bid BH3 complex, as well as KsBcl-2 residues 1–146 and Puma residues 130–155 in the KsBcl-2: Puma BH3 complex (Figure 2). The crystal structure of a KsBcl-2: Bid BH3 complex revealed that as previously seen for the related BHRF-1 and cellular Mcl-1, KsBcl-2 adopts a typical Bcl-2 fold with globular monomeric topology with eight alpha-helices and alpha-helices 2–5 form the canonical hydrophobic ligand binding groove.

**Table 1:** Binding affinities determined by Surface Plasmon Resonance of recombinant KsBcl-2 homolog KsBcl-2 with pro-apoptotic BH3 motif.

Peptide	IC_50_ (nM)
Bak	8 ± 0
Bax	16 ± 0
Bok	198 ± 13
Bad	NB
Bid	20 ± 9
Bik	64 ± 4
Bim	4 ± 1
Bmf	115 ± 10
Hrk	115 ± 10
Puma	26 ± 9

All BH3-peptides were 26-mers, except for a Bax BH3 28-mer and a Bid BH3 34-mer) peptides from human. All IC_50_ values (half maximal inhibitory constant in nM) are the means of three replicates with standard error. NB denotes no binding.

**Table 2:** X-ray diffraction data collection and refinement statistics.

	KsBcl-2: Bid BH3 (PDB ID: 7QTW)	KsBcl-2: Puma BH3 (PDB ID: 7QTX)
**Data Collection**		
Space group	P6_4_	C2
Cell dimensions		
a, b, c (Å)	89.48, 89.48, 51.06	59.23, 48.53, 57.39
α, β, γ (°)	90, 90, 120	90, 97.07, 90
Wavelength (Å)	0.9537	0.9537
Resolution (Å)	29.29–1.41 (1.46–1.41) *	32.46–2.11 (8.97–2.11) *
R_sym_ or R_merge_	0.12 (1.32) *	0.13 (0.81) *
I/σI	8.7 (0.2) *	4.2 (0.8) *
Completeness (%)	100.0 (99.6) *	98.1 (96.1) *
CC_1/2_	0.99 (0.30) *	0.98 (0.38) *
Redundancy	19.3 (11.5) *	2.4 (2.4) *
**Refinement**		
Resolution (Å)	29.29–1.41 (1.46–1.41) *	32.46–2.11 (8.97–2.11) *
No. reflections	44,973	9148
R_work_/R_free_	0.180/0.215	0.217/0.257
Clashscore	1.82	0.76
No. atoms		
Protein	1357	1310
Ligand/ion	40	15
Water	148	95
B-factors		
Protein	36.31	39.03
Ligand/ion	61.94	79.18
Water	49.45	42.92
R.m.s. deviations		
Bond lengths (Å)	0.013	0.002
Bond angles (°)	1.13	0.45

* Values in parentheses are for the highest resolution shell.

A structural analysis using DALI [48] showed that complexes of cellular Mcl-1 (PDB ID 3MK8) [44] and zebrafish pro-survival protein NRZ-1 (PDB ID 6FBX) [47] are the closest structural homologs of KsBcl-2 with RMSD values of 1.8 Å and 2.7 Å over 133 Cα and 138 Cα atoms with sequence identities of 20% and 17%, respectively. In comparison to other closely related viral Bcl-2 proteins including EBV BHRF1 (PDB ID 2V6Q) [45] and gammaherpesvirus 68 encoded M11 (PDB ID 3BL2) [26] gave an RMSD value of 2.6 Å and 2.8 Å over 128 Cα and 116 Cα atoms with significantly low sequence identities of 13% and 12% respectively. Interestingly, superimposition with fowlpox virus encoded Bcl-2 homolog FPV039 (PDB ID 5TZQ) [49] yields an RMSD value of 2.3 Å over 124 Cα atoms with a sequence identity of 16%. As expected, the backbone structure of KsBcl-2 is highly similar to previously characterized homologous counterparts. Binding of Bid BH3 peptide to KsBcl-2 occurs via the canonical ligand binding groove that is formed by helices α2–α5 (Figure 1a). The four canonical BH3 motif defining residues from Bid, I89, L93 and V96 and M100, are bound in four hydrophobic pockets of the ligand binding groove (Figure 2a). A fifth hydrophobic pocket in KsBcl-2 is occupied by Bid I85. The hallmark ionic interaction between pro-survival Bcl-2 proteins and pro-apoptotic BH3 motif ligands between a conserved arginine (R86^KsBcl-2^) in the BH1 motif and aspartate (D98^BID^) of the Bid BH3-motif [4] is also present. This interaction is supplemented by multiple hydrogen bonding interactions between Q82^BID^ and Q68^KsBcl-2^, I86^BID^ and S72^KsBcl-2^, D98^BID^ and N83^KsBcl-2^, D101^BID^ and G85^KsBcl-2^, R102^BID^ and Y140^KsBcl-2^ as well as between R102^BID^ and T146^KsBcl-2^ (Figure 2a). Similarly, in the KsBcl-2: Puma BH3 complex Puma residues I137, L141, M144 and L148 protrude into the four hydrophobic pockets and an extra hydrophobic pocket is occupied by Puma residue W133 (Figure 2d). Additionally, an ionic interaction is formed by R142^PUMA^ and D79^KsBcl-2^. However, the expected ionic interaction between pro-survival Bcl-2 proteins and pro-apoptotic BH3 motif ligands between a conserved arginine in the BH1 motif and aspartate of the BH3-motif [4] was surprisingly absent. Additional hydrogen bonds are found between Q132^PUMA^ and Q68^KsBcl-2^, R142^PUMA^ and V76^KsBcl-2^, N149^PUMA^ and N83^KsBcl-2^ as well as between N149^BID^ and Y140^KsBcl-2^ (Figure 2d).

**Table 3:** Summary of pro-apoptotic BH3 motif affinities for KsBcl-2 and its closely related homologs.

Affinity (nM)						
Peptide	KsBcl2 ^b^ (This Study)	BHRF-1 ^a^ [45]	M11 ^a^ [26]	Mcl-1 ^b^ [46]	NRZ ^a^ [47]	FPV039 ^a^ [49]
Bak	8	150	76	10	N/A	76
Bax	16	1400	690	12	688	76
Bok	198	N/A	N/A	N/A	N/A	N/A
Bad	NB	NB	N/A	>100,000	343	653
Bid	20	110	232	2100	409	2
Bik	64	NB	N/A	1700	12	30
Bim	4	18	131	2.4	41	10
Bmf	NB	NB	300	1100	NB	254
Hrk	115	NB	719	370	N/A	24
Noxa	220	NB	132	24	142	28
Puma	26	70	370	6.3	36	31

N/A-Not Applicable, NB-No Binding. ^a^—Affinity measured in nM by Isothermal Titration Calorimetry. ^b^—Affinity measured in nM by SPR.

## 4. Discussion

Bcl-2 homologs are widely used amongst large DNA viruses to ensure viral proliferation and/or survival [5,20,50]. Bcl-2 homologs have been described for members of the families of *herpesviridae*, *poxviridae* [51,52,53], *asfaviridae* [54] and *iridoviridae* [55]. Amongst the *herpesviridae*, the majority of *gammaherpesvirinae* genera have been shown to encode apoptosis inhibiting Bcl-2 homologs including EBV [45,56], KSHV, murine gammaherpesvirus 68 and herpesvirus saimiri [14]. Whilst many *gammaherpesvirinae* encode for sequence, structural or functional pro-survival Bcl-2 homologs, considerable sequence, structural and affinity profile diversity exists amongst these proteins [50]. There are widely differing interaction profiles with host proapoptotic Bcl-2 proteins and some differences in overall structure as well as detailed interactions at the atomic level, as might be expected from these highly sequence divergent Bcl-2 fold sequences [5]. Here, we report that the *gammaherpesvirinae* member KSHV Bcl-2 (KsBcl-2) is a potentially potent inhibitor of mammalian apoptosis. KsBcl-2 adopts a globular monomeric Bcl-2 fold (Figure 1a) and binds a number of BH3 motif peptides from human pro-apoptotic Bcl-2 proteins including those from Bax, Bak, Bok, Bim, Bid, Bik, Noxa, Puma and Hrk (Table 1) but we did not detect any affinity for Bad or Bmf. Among the cellular Bcl-2 proteins, Bcl-x_L_, Bcl-2 and Bcl-w bind a significant number of BH3-only proteins but show no affinity towards Noxa [4,32]. The previously characterized viral Bcl-2 proteins, tanapox virus TANV16L [53], Sheeppox virus SPP14 [57,58], fowlpox virus FPV039 [49], African swine fever virus A179L [54,59] and murine gammaherpesvirus M11 [26] were able to bind a substantial number of BH3-only proteins. KsBcl-2 displays the tightest binding to the key executioner Bcl-2 proteins Bax and Bak amongst vBcl-2 proteins examined to date with affinities that are comparable to human pro-survival Bcl-2 proteins such as Mcl-1 [46]. However we note that African swine fever virus A179L is nearly as potent a binder of Bak and Bax [54]. In contrast, other herpesviral vBcl-2 proteins, such as EBV BHRF1 (human herpes virus 4) [45] and murine herpesvirus 68 M11 proteins [26], harbor a more restricted overall pro-apoptotic Bcl-2 binding profile and bind Bak and Bax more weakly.

**Figure 3:**
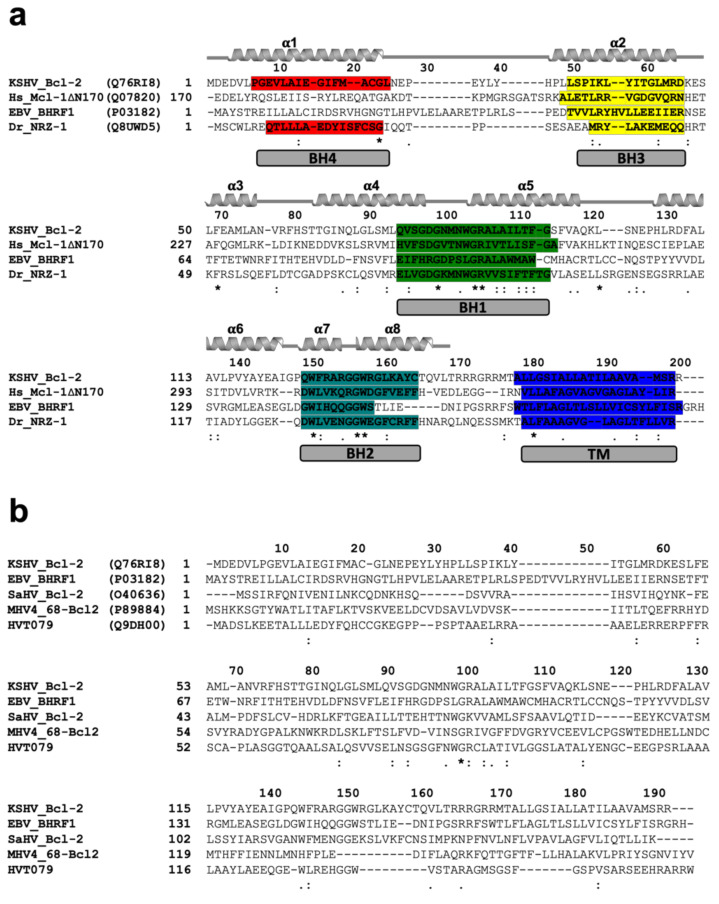
Sequence alignments (**a**) Sequence alignment of KsBcl-2 with pro-survival Bcl-2 family members. The sequences of KSHV KsBcl-2 (UniProt accession number Q76RI8), human Mcl-1 (UniProt Q07820) EBV BHRF1 (UniProt P03182), Zebrafish pro-survival Bcl-2 protein NRZ1 (UniProt Q8UWD5) and were aligned using muscle [42]. Secondary structure elements are marked based on the crystal structure of KsBcl-2, and BH motifs are highlighted in colours: BH4, red; BH3, yellow; BH1, green; BH2, teal and trans-membrane region (TM) in blue. The regions of helix are marked and unstructured loops with a bar below the sequence, conserved residues are denoted by ‘*’, with highly conservative substitutions indicated by ‘:’ and conserved substitutions indicated by ‘.’. (**b**) Sequence alignment of pro-survival Bcl-2 proteins encoded by different herpes viruses. The sequences of KSHV KsBcl-2 (UniProt Q76RI8), EBV BHRF1 (UniProt P03182), Human Saimiri virus Bcl-2 protein 16L (HVS) (UniProt O40636), Murine gammaherpes virus 68 Bcl-2 protein, M11(UniProt: P89884) and Turkey herpes virus Bcl-2 protein HVT079 (UniProt: Q9DH00). Conserved residues, highly conservative residues and conserved residues are indicated as in (**a**).

Interestingly, our structural homolog search using DALI [48] identified the closest structural homolog to KsBcl-2 as human Mcl-1 (PDB ID 3MK8) [44]. KsBcl-2 shares 21% sequence identity and structural superimposition of KsBcl-2 with Mcl-1 yielded an R.M.S.D value of 1.8 Å over 133 Cα atoms. The closest viral Bcl-2 homolog identified was fowlpox virus protein, FPV039 [49] with a sequence identity of 16% and structural superimposition yielding an R.M.S.D value of 2.3 Å over 130 Cα atoms. Sequence alignment of KsBcl-2 (Figure 3) with its closest structural homologs showed that KsBcl-2 shares sequence features of other multi-domain members of the Bcl-2 family and shares 24% sequence identity with NRZ, the zebrafish Bcl-2 protein [47], 17% identity with EBV BHRF1 [45]. Similarly, sequence alignment of KsBcl-2 with herpes virus encoded Bcl-2 homolog produces 19% identity for herpes virus saimiri encoded Bcl-2 homolog 16L (HVS 16L) [14], 10% identity for murine gammaherpes virus 68 Bcl-2 homolog M11 [17] and 27% identity for turkey herpes virus Bcl-2 homolog HVT079 [15,16]. However, the BH regions show a significantly lower level of conservation among these proteins [60] and these sequence variations that are located in the ligand binding groove create the basis in part for the selectivity differences observed for BH3-ligands compared to the cellular counterparts. Superimposition of KsBcl-2 with EBV BHRF1 produced an R.M.S.D of 2.8 Å over 83 residues whereas gammaherpesvirus 68 encoded M11 produced an R.M.S.D of 10.2 Å over 42 residues. EBV BHRF1 is very similar in structure to KsBcl-2, whereas M11 appears to be significantly different.

Phasing of our crystal structures relied on a model generated using AlphaFold [37]. Superimposition of the experimentally determined structure compared with the AlphaFold model yielded an RMSD of 0.7 Å. The alpha fold model featured nearly identical side chain rotamers and secondary structure elements, with some minor variations in the alpha 3 helix. However, significant variation can be seen in the unstructured N-terminal end and the loop region between alpha 1–2 helices, which accounts for the overall RMSD difference observed.

A comparison of overall fold of the KsBcl-2: Bid BH3 complex reveals that it is very similar to that observed in other Bcl-2: BH3 complexes. Despite the overall similarity in fold, several key differences are observed in the crystal structures and protein: peptide interfaces of the KsBcl-2: Bid and Mcl-1: Bid (PDB ID: 2KBW) [61] complexes.

**Figure 4:**
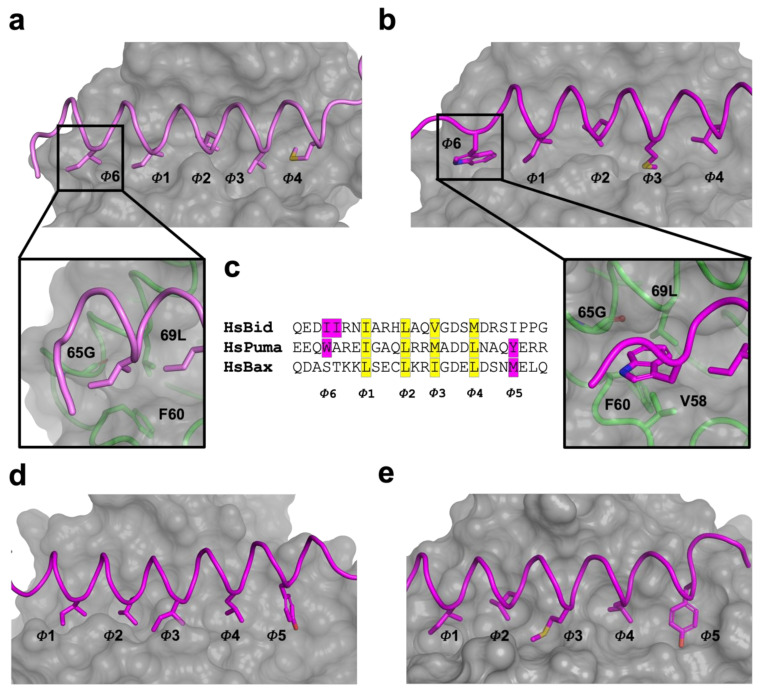
Engagement of an additional hydrophobic pocket (fifth pocket) in viral Bcl-2 proteins with BH3motif peptides. (**a**) Hydrophobic binding groove of KsBcl-2:Bid BH3 complex. The hydrophobic pockets of the binding groove are labelled as *Φ*1–*Φ*6. The surface of the KsBcl-2 is shown in grey with side chains of the key hydrophobic residues located in the pocket *Φ*1–*Φ*6 shown as sticks. Bid BH3 backbone is shown as cartoon tube (pink). An enlarged view of the fifth hydrophobic pocket of KsBcl-2 is shown in the bottom panel with a cartoon tube of the KsBcl-2 backbone (green), with residues involved in forming the fifth hydrophobic pocket labelled. (**b**) Hydrophobic binding groove of KsBcl-2:Puma BH3 complex. Puma BH3 backbone is shown as cartoon tube (magenta). The surface, hydrophobic pockets of the binding groove and key residues are shown as in (**a**). (**c**) sequence of the key BH3 motif peptides (Bid, Puma and Bax) involved to engage fifth hydrophobic pocket with viral Bcl-2 proteins are shown. Conserved four hydrophobic residues engage with hydrophobic pockets are highlighted in yellow and labelled *Φ*1–*Φ*4. The additional hydrophobic residues engaging with the fifth hydrophobic pocket are highlighted in magenta and labelled as *Φ*5 (C-terminal pocket) and *Φ*6 (N-terminal pocket). (**d**) Hydrophobic binding groove of Mcl-1:Puma BH3 complex. Puma BH3 (magenta) backbone is shown as cartoon tube. (**e**) Hydrophobic binding groove of ORFV125:Puma BH3 complex. Puma BH3 (magenta) backbone is shown as cartoon tube. The surface, hydrophobic pockets of the binding groove and key residues are shown as in (**a**).

A comparison of structure and interactions of KsBcl-2:Puma (Figure 2d) complex reveals it is nearly identical to those observed in Mcl-1:Puma complex (Figure 2e) or ORFV125:Puma [62] complex (Figure 2f) whereas Mcl-1:Puma interaction is four-fold tighter than KsBcl-2:Puma and ORFV125: Puma interaction is approximately 60-fold weaker than KsBcl-2:Puma [63]. KsBcl-2 binding of either Puma BH3 or Bid BH3 utilizes an additional hydrophobic pocket from the base of the binding groove. For the KsBcl-2:Bid complex this additional hydrophobic pocket is utilized by N-terminal residue I85^BID^ (Figure 2a and Figure 4a) and in KsBcl-2:Puma complex this additional hydrophobic pocket is utilized by W133^PUMA^ (Figure 2b and Figure 4b). In comparison to previously reported viral Bcl-2: BH3 interactions, only three of the BH3 peptides (Bax, Bid and Puma (Figure 4c)) were shown to have this fifth pocket. A similar observation was noted previously for the variola virus VARV F1L:Bid complex (Figure 2c) [64]. However, this fifth hydrophobic pocket in the KsBcl-2 complexes is different to that observed for other complexes. For instance, both Mcl-1:Puma (Figure 4d) [65], ORFV125:Puma (Figure 4e) [62], SPPV14:Bax [58] and ORFV125:Bax [63] complexes, the C-terminal residue Y142^PUMA^ and M74^BAX^ utilized the fifth pocket respectively. Whereas, the fifth hydrophobic pocket is absent in both TANV16L:Puma or TANV16L:Bax complexes [53]. Furthermore, Puma is crucial for p53-independent and dependent apoptosis regulation against diversified stimuli [66], such as viral infection, dysregulated oncogene expression, radiation induced apoptosis, genotoxic stress and toxins. In contrast to other BH3-only proteins, Puma interacts with almost all viral Bcl-2 proteins [50] as well as five of the major cellular Bcl-2 proteins such as Bcl-x_L_, Bcl-2, Mcl-1, Bcl-w and A1 with high affinity interactions [67]. The combination of structural data and conserved interactions between Puma BH3 and pro-survival Bcl-2, BH3-mimetic drugs or selective peptide inhibitors against KsBcl-2 [68] was used to mimic these interactions [69].

The BH1 motif of multi-domain Bcl-2 family proteins, along with pro-apoptotic Bcl-2 protein Bak, Bax and Bok plays a crucial structural role during cell death regulation and can be easily recognized by a short well conserved signature sequence motif “NWGR” in the BH1 motif at the N-terminal end the α5 helix [7] (Figure 4). The Gly and Arg residues of ‘NWGR” motif are important for protein–protein interactions within the Bcl-2 family proteins [65,70,71]. The Arg residue is located at the beginning of the α5 helix forms the canonical ionic/salt bridge interaction with the conserved Asp residue of BH3 motif of the pro-apoptotic Bcl-2 proteins. In the KsBcl-2: Bid complex, the corresponding R86^KsBcl^^-2^ residue in the NWGR motif interacts with D98 of the Bid BH3 peptide. However, this highly conserved interaction was not seen or is very weak in the KsBcl-2: Puma complex where the closest proximity for this interaction is 5.6 Å. In place of this interaction an alternative salt bridge is observed between D79 ^KsBcl-2^ and R142^Puma^. An identical ionic interaction can be seen in Mcl-1: Puma complex [65] where D256^Mcl-1^ is conserved in both KsBcl-2 and Mcl-1. In contrast, human Mcl-1: Bid (PDB ID: 2KBW) [61] complex did not feature the canonical salt-bridge interaction, whereas the human Mcl-1: Puma complex formed this interaction between corresponding R263^Mcl-1^ residue in the NWGR motif interacts with D146 of the Puma BH3 peptide. Previous studies showed that a R263A^Mcl-1^ mutation in Mcl-1 results significant loss of ability to interact with BH3 peptide [72]. Comparison of the binding mode of Bid BH3 or Puma BH3 with KsBcl-2 identified three structurally conserved residues: Q68, N83 and Y140 in the binding groove which make polar contacts with the BH3 motif of pro-apoptotic Bcl-2 proteins. This suggests that KsBcl-2 residues Q68, N83 and Y140 are important for stabilization of the BH3 motif peptide in the binding groove.

Considering that Mcl-1, KsBcl2 and ORFV125 feature highly similar binding site (Figure 4) it is interesting that they feature significantly differing affinities for Puma. Mcl-1:Puma features the hallmark interaction between the conserved Arg residue of the NWGR motif and the well conserved Asp residue of BH3 motif, whereas in KsBcl-2:Puma the NWGR motif is present but this hallmark interaction with the conserved Asp from the BH3 motif is absent. This may contribute to the four-fold decrease in affinity of KsBcl-2:Puma complex compared to that observed for the Mcl-1:Puma complex. The equivalent BH1 region of ORFV125 features the sequence “SPGR” instead of the conserved NWGR and displays an approximately 250-fold reduced affinity against Puma BH3. Thus, the “SPGR” sequence motif of ORFV125 is directly impacting specificity and binding compared to KsBcl-2.

KsBcl-2 displays a very broad ligand binding profile when compared to its closest homologous proteins, Mcl-1, NRZ, and FPV039, BHRF1 and M11 (Table 3), even when taking into consideration that different methods were used to determine these affinities. However, KsBcl-2 is the only protein that interacts with BH3 motif peptide of pro-apoptotic protein Bok and showed very strong affinity towards Bak, Bax, Bim, Bid, Puma and Bik BH3 motif peptides. Previous studies revealed that overexpression of KsBcl-2 efficiently blocked host cell apoptosis similar to that observed in cellular Bcl-2, Bcl-x_L_, Mcl-1 or EBV (gammaherpes virus 4) encoded BHRF1 but was not able to heterodimerized with Bax or Bak [13], suggesting that KsBcl-2 may have developed a strategy to escape any negative regulatory effect from cellular Bak and Bax. Overexpression of KsBcl-2 is sufficient to block mitochondrial outer membrane permeabilization and subsequent apoptosis during latent B-cell infection [68]. Overall KsBcl-2 displays a ligand binding profile that most closely resembles Mcl-1, albeit with varied affinities for individual ligands.

Interestingly, KsBcl-2 was shown to be involved in virion assembly via tegument protein (ORF55), with a reported interaction via its N-terminal amino acid residue 11–20 [30]. In our crystal structures and the previously reported NMR structure [73] KsBcl-2 adopts a helical configuration in the N-terminal region proposed as the interacting site, and crucially the identified Glu14 residue is solvent exposed and available for an interaction. However, the precise mode of engagement of ORF55 with KsBcl-2 remains to be clarified and likely requires structure determination of such a complex.

In summary, we report the biochemical and structural analysis of Kaposi sarcoma herpes virus encoded apoptosis inhibitor KsBcl-2, which revealed a broad high affinity binding profile for mammalian pro-apoptotic Bcl-2 proteins. Our findings provide a molecular basis for dissecting the function of KsBcl-2 in KSHV infection and to determine the contribution that abolition of Bcl-2 mediated apoptosis makes to the KSHV life cycle.

## Data Availability

Data supporting the findings of this manuscript are available from the corresponding authors upon reasonable request. Coordinate files were deposited at the Protein Data Bank (https://www.rcsb.org/) (accessed on 1 July 2021) using accession codes 7QTW and 7QTX for KsBcl-2: Bid BH3 and KsBcl-2: Puma BH3, respectively. The raw X-ray diffraction data were deposited at the SBGrid Data Bank [41] (https://data.sbgrid.org/data/) using their PDB accession codes 7QTW and 7QTX for KsBcl-2: Bid BH3 and KsBcl-2: Puma BH3, respectively.
